# Durability of Protection Post–Primary COVID-19 Vaccination in the United States

**DOI:** 10.3390/vaccines10091458

**Published:** 2022-09-03

**Authors:** Amanda Zheutlin, Miles Ott, Ran Sun, Natalia Zemlianskaia, Craig S. Meyer, Meagan Rubel, Jennifer Hayden, Breno Neri, Tripthi Kamath, Najat Khan, Sebastian Schneeweiss, Khaled Sarsour

**Affiliations:** 1 Data Sciences, Research & Development, Janssen Pharmaceuticals, Titusville, NJ 08560, USA; 2 Division of Pharmacoepidemiology, Department of Medicine, Brigham and Women’s Hospital and Harvard Medical School, Boston, MA 02120, USA

**Keywords:** vaccine effectiveness, Ad26.COV2.S, BNT162b2, mRNA-1273, durability

## Abstract

The durability of immune responses after COVID-19 vaccination will drive long-term vaccine effectiveness across settings and may differ by vaccine type. To determine durability of protection of COVID-19 vaccines (BNT162b2, mRNA-1273, and Ad26.COV2.S) following primary vaccination in the United States, a matched case-control study was conducted in three cohorts between 1 January and 7 September 2021 using de-identified data from a database covering 168 million lives. Odds ratios (ORs) for developing outcomes of interest (breakthrough SARS-CoV-2 infection, hospitalization, or intensive care unit admission) were determined for each vaccine (no direct comparisons). In total, 17,017,435 individuals were identified. Relative to the baseline, stable protection was observed for Ad26.COV2.S against infections (OR [95% confidence interval (CI)], 1.31 [1.18–1.47]) and hospitalizations (OR [95% CI], 1.25 [0.86–1.80]). Relative to the baseline, protection waned over time against infections for BNT162b2 (OR [95% CI], 2.20 [2.01–2.40]) and mRNA-1273 (OR [95% CI], 2.07 [1.87–2.29]) and against hospitalizations for BNT162b2 (OR [95% CI], 2.38 [1.79–3.17]). Baseline protection remained stable for intensive care unit admissions for all three vaccines. Calculated baseline VE was consistent with published literature. This study suggests that the three vaccines in three separate populations may have different durability profiles.

## 1. Introduction

Clinical trials have demonstrated high vaccine efficacy of the primary series in protection against Coronavirus disease 2019 (COVID-19) for all three US-authorized or approved vaccines (Ad26.COV2.S, BNT162b2, and mRNA-1273) [1,2,3], and subsequent studies have confirmed high vaccine effectiveness (VE) against infection and severe disease in the real-world [4,5,6,7,8]. Preliminary data on waning VE and the emergence of severe acute respiratory syndrome coronavirus 2 (SARS-CoV-2) variants of concern have prompted regulatory approval or emergency use authorization for booster doses in the United States for all adults. Subsequently, the Centers for Disease Control and Prevention (CDC) recommended that BNT162b2 or mRNA-1273 are preferred to Ad26.COV2.S.

Studies on the durability of all three US vaccines in the general population have been limited to date, with many focusing on high-risk populations or specific geographic areas [7,9,10,11,12,13]. Durability of responses to primary vaccine regimens remains especially important for the management of the pandemic globally, as much of the world remains unvaccinated or only recently vaccinated with the primary series. As such, it is critical to assess the degree to which protection is sustained for each vaccine over time in the general adult population. In this study, we report the durability of protection against COVID-19 in over 17 million vaccinated individuals following primary vaccination separately for each approved or authorized vaccine against breakthrough infections, hospitalizations, and intensive care unit (ICU) admissions, while accounting for calendar time, residential location, age, sex, and comorbid conditions.

## 2. Materials and Methods

### 2.1. Study Design

We used a matched case-control design conducted with three cohorts of vaccinated individuals to assess the durability of vaccine-induced protection against SARS-CoV-2 infections, COVID-19-related hospitalizations, and COVID-19-related ICU admissions in the United States between 1 January and 7 September 2021, as per Ray and Klein [14,15]. The three vaccines were authorized at different times resulting in differences in both vaccination and follow-up time, as well as demographics (e.g., older individuals were eligible for vaccination prior to authorization of Ad26.COV2.S). Our main analysis required full vaccination to occur on after 27 February 2021, the first date at which all three vaccines were available, such that the second mRNA (BNT162b2 and mRNA-1273 cohorts) vaccine dose and the first (and only) dose for the Ad26.COV2.S cohort was on or after 27 February 2021. We also analyzed all available data for the mRNA vaccine cohorts where a first mRNA dose was on or after 1 January 2021 (Figure S1 for all cohort construction).

We compared the odds of breakthrough infection, hospitalization, or ICU admission in each subsequent 28-day window (months 2 through 6+ since vaccination) relative to the first month since vaccination (reference month lasting 28 days). Here, durability was defined as stable odds for the outcome of interest across time. Follow-up started after a 21-day period following the final vaccination dose; day 22 is thus the beginning of the first month of follow-up (Figure S1). We selected this timeframe to allow for the 14 days required for full vaccination and an additional 7-day washout period to ensure COVID-19-related outcomes included in our study were limited to those due to COVID-19 infections acquired after completion of the primary vaccination [16]. Since the CDC guidelines recommend waiting 5 to 7 days after exposure before being tested [17], test results and hospitalizations recorded soon after vaccination (i.e., 15–21 days after the last dose) may reflect infections and exposures occurring prior to vaccination.

Data are publicly available and were de-identified according to the Health Insurance Portability and Accountability Act patient confidentiality requirement and did not qualify as human subjects research under the Common Rule, and therefore not subject to review by an institutional review board [18]. The Advarra Institutional Review Board, an Ethically Competent Body, approved this exemption (no.Pro00060389).

### 2.2. Population

We defined eligible individuals as adults aged 18 years or older who were vaccinated following the recommended primary vaccination schedule (Supplementary Methods). For mRNA-1273, two doses 28 to 42 days apart were required; for BNT162b2, two doses 21 to 42 days apart were required; and for Ad26.COV2.S, one dose was required [19]. For cohort analyses using data available from 27 February 2021 to 7 September 2021, we required the second dose of mRNA-1273 or BNT162b2 and the first dose of Ad26.COV2.S to be on or after 27 February 2021, the earliest point when all three were available. For mRNA cohorts using all available data, we included individuals with a first dose of mRNA-1273 or BNT162b2 on 1 January 2021, or later. Cohort entry began 21 days after the last vaccine dose (Figure S1). Cases for each outcome were matched to eligible vaccinated patients without the respective event of interest within each of the separate vaccine cohorts. Further details for exclusion criteria, and the selection of cases and controls are described in Supplementary Methods.

### 2.3. Exposure

The exposure of interest was the time in months from cohort entry to the outcome of interest. The first outcome of interest was infection identified by either a COVID-19 ICD-10-CM diagnosis code (U07.1, U07.2) or a positive polymerase chain reaction (PCR) laboratory test. Incident hospitalization and ICU admission were included as additional outcomes to index more severe disease. We grouped follow-up time (occurring between 21-days post completed vaccination and 7 September 2021) into five exposure categories: month 1 or reference month (1–28 days after follow-up started), month 2 (29–56 days), month 3 (57–84 days), month 4 (85–112 days), month 5 (113–140 days), and month 6+ (141 or more days).

### 2.4. Statistical Analysis

To assess the durability of protection, we fitted conditional logistic regression models separately for each vaccine and outcome combination, resulting in nine models. We estimated the effect of each additional month after receipt of the primary series vaccine dose on the odds of the outcome of interest using month 1 as the reference category. Odds ratios (ORs) with 95% confidence intervals (CIs) measured change in baseline protection relative to the referent month.

As ORs here reflect change in risk over time for each vaccine cohort separately (and thus estimate change in risk from different baseline groups), we translated ORs for infection and hospitalization to VE (see Supplementary Methods) by incorporating all eligible vaccinated individuals (not restricted to case-control matched groups) and unvaccinated individuals. Sensitivity analyses stratifying by age and comorbidity score were also conducted (see Supplementary Methods).

## 3. Results

### 3.1. Vaccine Cohorts, Cases, and Controls

Individuals fully vaccinated with one of the three authorized or approved vaccines in the United States and meeting study inclusion and exclusion criteria represented the broader vaccinated cohorts from which our matched case-control samples were drawn (see attrition flowchart, Figure S2). Together, 17,017,435 individuals were included in these cohorts, representing close to 10% of all individuals fully vaccinated in the United States by the end of our study period, 7 September 2021 [20]. Overall vaccine cohorts appeared to differ in some demographic factors across vaccines (Table 1), reflecting variation in vaccine authorization and eligibility timing, as well as distribution and availability. Among those vaccinated on or after 27 February 2021, mean age was older in mRNA-1273 (55.3 years) relative to Ad26.COV2.S (50.9 years) and BNT162b2 (50.3 years). More individuals had high (≥2) comorbidity scores in mRNA-1273 (12.4%) than BNT162b2 (10.6%) and Ad26.COV2.S (10.5%), as well. Ad26.COV2.S had fewer women and more individuals insured through Medicaid (52% and 8.7%, respectively) than BNT162b2 (57% and 7.2%) or mRNA-1273 (57% and 7.3%). The number of cases and matched controls for each outcome for each vaccine are listed in Table S1. Across vaccines and outcomes, the included cases represented 98% of all eligible cases. Compared to those vaccinated on or after 27 February, cases among those who were vaccinated earlier (from 1 January to 27 February) had substantially more comorbidities (Gagne score ≥ 4, 26.1% vs. 7.3%) and were older (age ≥ 85, 28.7% vs. 3.2%; Table S2).

### 3.2. Change in Effectiveness of Ad26.COV2.S over Time

**Figure 1:**
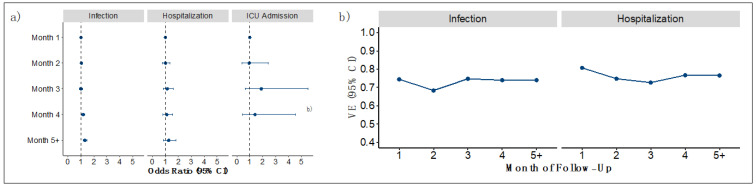
Ad26.COV2.S estimated durability and vaccine effectiveness from 27 February 2021 to 7 September 2021. (**a**) Estimated ORs (95% CI) assessing durability of baseline vaccine protection against infections, hospitalizations, and ICU admissions; (**b**) estimated VE (95% CI) against infections and hospitalizations by month of follow-up for primary vaccination.

Among those who were vaccinated with the Ad26.COV2.S vaccine, waning of protection against infection was observed in month 5+ with a statistically significant 31% higher odds of infection compared to the baseline (OR [95% CI], 1.31 [1.18–1.47]; Figure 1a and Table S3). Corresponding calculated VE was stable across the study period. Calculated VE was 74% (95% CI, 72–75) at month 1 and 74% (95% CI, 70–76) at month 5 or any time after (Figure 1b and Table S4). The odds of hospitalization increased by 25% in month 5+ compared to the baseline but was not statistically significant (OR for month 5+ = 1.25 [95% CI, 0.86–1.80]). Translated VE ranged from 81% (95% CI, 76–82) at month 1 to 77% (95% CI, 64–83) at month 5 or after. For ICU admission, point estimates were non-monotonic over time and non-significant suggesting protection against ICU admission did not wane over time. Similarly, when stratifying by age or comorbidity score, stable protection against infection and hospitalization was observed across strata (Figures S3–S6).

### 3.3. Change in Effectiveness of BNT162b2 over Time

**Figure 2:**
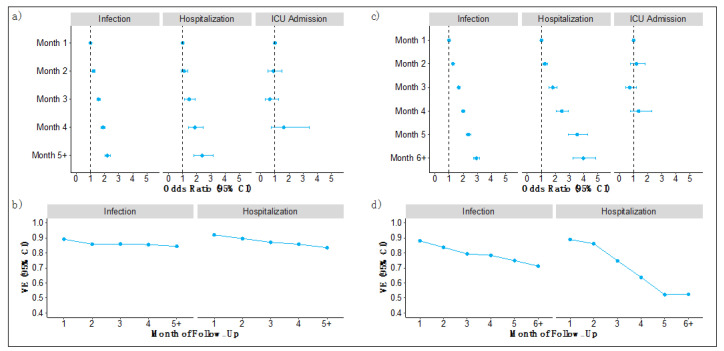
BNT162b2 vaccine durability and vaccine effectiveness using two available time periods in (**a**,**b**) from 27 February 2021 to 7 September 2021; and (**c**,**d**) from 1 January 2021 to 7 September 2021. Each time period presents separately (**a**,**c**) estimated ORs (95% CI) assessing durability of baseline vaccine protection against infections, hospitalizations, and ICU admissions; (**b**,**d**) estimated VE (95% CI) against infections and hospitalizations by month of follow-up for primary vaccination.

Among those vaccinated with BNT162b2 dose 2 on or after 27 February 2021, the odds of a breakthrough infection similarly increased each month of follow-up with a 120% higher odds of infection in month 5+ (OR for month 5+ of BNT162b2 = 2.20 [95% CI, 2.01–2.40]; Figure 2a and Table S3) and corresponding translated VE ranged from 89% (95% CI, 88–89) at month 1 to 84% (95% CI, 82–85); Figure 2b and Table S4). For hospitalizations, odds increased monotonically (OR for month 5+ = 2.38 [95% CI, 1.79–3.17]) and translated VE ranged from 92% (95% CI, 91–93) at month 1 to 84% (95% CI, 79–88) at month 5 or after. In age- and comorbidity-stratified groups, protection against infection and hospitalization protection waned similarly in all groups and was consistent with the effects observed in the overall BNT162b2 cohort (Figures S3–S6).

Among all those vaccinated with BNT162b2 (dose 1 ≥ 1 January 2021), the odds of a breakthrough infection were statistically significant and increased monotonically each month of follow-up with 193% higher odds of infection in month 6+ (OR for month 6+ of BNT162b2 = 2.93 [95% CI, 2.72–3.15]; Figure 2c and Table S3) and translated VE ranged from 88% (95% CI, 87–88) at month 1 to 71% (95% CI, 69–73) at month 6 or after (Figure 2d and Table S4). Odds of hospitalization increased monotonically with a statistically significant 297% higher odds of hospitalization in month 6+ (OR for month 6+ = 3.97 [95% CI, 3.26–4.83]) and VE against hospitalization ranged from 89% (95% CI, 88–90) at month 1 to 52% (95% CI, 43–61) at month 6 or after. Odds ratios for ICU admission did not suggest waning protection at any point.

### 3.4. Change in Effectiveness of mRNA-1273 over Time

**Figure 3:**
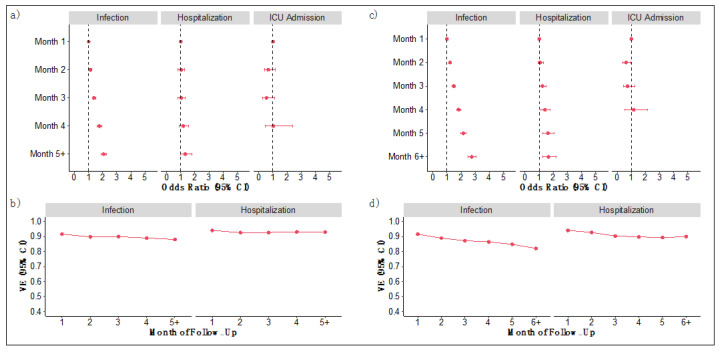
mRNA-1273 vaccine durability and vaccine effectiveness using two available time periods in (**a**,**b**) from 27 February 2021 to 7 September 2021; and (**c**,**d**) from 1 January 2021 to 7 September 2021. Each time period presents separately (**a**,**c**) estimated ORs (95% CI) assessing durability of baseline vaccine protection against infections, hospitalizations, and ICU admissions; (**b**,**d**) estimated VE (95% CI) against infections and hospitalizations by month of follow-up for primary vaccination.

Among those vaccinated with the mRNA-1273 vaccines dose 2 ≥ 27 February 2021, the odds of a breakthrough infection also increased monotonically for each month of follow-up with a statistically significant 107% higher odds of infection in month 5+ compared to the baseline (OR for month 5+ of mRNA-1273 = 2.07 [95% CI, 1.87–2.29]; Figure 3a and Table S3). Corresponding VE against infection ranged from 92% (95% CI, 91–92) at month 1 to 88% (95% CI, 87–89) at month 5 or after (Figure 3b and Table S4). Although the odds of hospitalization were 32% higher in month 5+ compared to the baseline (OR for month 5+ = 1.32 [95% CI, 0.98–1.79]), odds ratios for hospitalizations were not statistically significant and translated VE was stable ranging from 94% (95% CI, 93–95) at month 1 to 93% (95% CI, 91–95). In age- and comorbidity-stratified groups, protection against infection and hospitalizations, modest waning was observed in higher-risk groups, but not in lower-risk groups (Figures S3–S6).

Among all those vaccinated with mRNA-1273 (dose 1 ≥ 1 January 2021), odds of a breakthrough infection were statistically significant and increased monotonically with a 176% higher odds of infection in month 6+ compared to the baseline (OR for month 6+ of mRNA-1273 = 2.76 [95% CI, 2.51–3.04]; Figure 3c and Table S3) and corresponding translated VE against infection ranged from 92% (95% CI, 91–92) at month 1 to 82% (95% CI, 80–83; Figure 3d and Table S4). Odds of hospitalization were similarly statistically significant and increased monotonically each month with a 66% higher in month 6+ compared to the baseline (OR for month 6+ = 1.66, 95% CI [1.26–2.19]) and translated VE ranged from 94% (95% CI, 93–95) at month 1 to 90% (95% CI, 87–92). Odds ratios were non-monotonic and non-significant for COVID-19 related ICU admission suggesting protection was stable.

## 4. Discussion

Using claims data for over 168 million individuals in the United States from 1 January to 7 September 2021, we used a matched case-control study design to assess the durability of protection against breakthrough infections, hospitalizations, and ICU admissions over time from full vaccination. The database used in this study was representative of the proportions of payer type used by the broader US population [20,21]. Although unable to distinguish between declining effectiveness due to immunological processes or the emergence of novel variants, the study provides insights into real-world outcomes of vaccines over time. By matching cases and controls for each vaccine on confounding variables including calendar time and location, any impact of viral strain and the variable background rates of infection would likely be accounted for [22]. The study was designed to compare each vaccine to itself and therefore direct comparison between vaccines should be avoided, as no formal matching or adjustment for baseline differences or analysis was made between vaccines.

Although, as seen in randomized trials, the level of efficacy of a single-dose Ad26.COV2.S vaccine was lower than that seen with two-dose mRNA vaccines, we found that the level of protection after vaccination with the Ad26.COV2.S vaccine suggested consistent durability relative to baseline risk for both breakthrough infections and hospitalizations. For both mRNA vaccines, protection against infections appeared to wane in all considered cohorts and sub-groups (age- and comorbidity-stratified) relative to their baseline risk. The magnitude of loss of durability appeared larger for mRNA cohorts allowing vaccinations from 1 January 2021 compared to those vaccinated on or after 27 February 2021, potentially reflecting differences in high-risk groups with early vaccine prioritization (see Table 1). Protection against hospitalization was generally stable for mRNA-1273 in younger, healthier cohorts, and waned modestly in higher-risk groups. For BNT162b2, protection against hospitalization similarly appeared to wane in all cohorts and sub-groups. All vaccines suggested durable protection over the study period against ICU admission as all estimates of association over time were non-monotonic and non-significant with confidence intervals containing the null value, showing sustained protection against critically severe disease.

Recent studies have suggested a more stable neutralizing antibody response for those inoculated with the Ad26.COV2.S vaccine as compared to the BNT162b2 and mRNA-1273 vaccines, potentially due to differences in the mechanism of action [23,24], which may translate into more durable protection. Results from this study corroborate this deduction. However, any statement about the durability of VE should be interpreted with caution due to potential floor or ceiling effects where a more effective vaccine has more room for waning compared to a less effective one. Additionally, durability of protection is only one feature of each vaccine and must be considered together with other characteristics, including baseline effectiveness, when assessing overall protection.

A strength of our study design comparing the odds of outcome events from later periods to a period soon after full vaccination is that it avoids the need for an unvaccinated control group, which in national insurance claims databases may be subject to confounding and underreporting [4]. However, as the observed ORs can be difficult to interpret, we incorporated assumptions about the incidence rate in the unvaccinated population during the study period, and indirectly estimated baseline and subsequent VE for each vaccine and outcome (Supplementary Methods). The measure of VE allowed us to address limitations with measures of relative risk by standardizing our estimates of risk against a common unvaccinated population. The strength of this approach is that the VE estimates provide context to the magnitude of the odds ratios observed in the study relative to the baseline risk. Our translated baseline VE estimates were consistent with the published literature for the three vaccines primary series showing mRNA-1273 to have the highest level of protection, followed by BNT162b2, and by single-dose Ad26.COV2.S, adding face validity to the method and assumptions of the VE translation [1,2,3,4,5,6,7,8,25].

Studies specifically examining durability of VE have been limited. A recent meta-analysis of studies evaluating durability defined as change over time in VE identified 78 studies (BNT162b2, n = 38; mRNA-1273, n = 23; Ad26.COV2.S, n = nine; and AstraZeneca-Vaxzevria, n = eight) [26]. The study concluded that, on average for all four vaccines, vaccine efficacy or effectiveness for symptomatic COVID-19 decreased by 24.9% (95% CI, 13.4–41.6) in people of all ages. For severe COVID-19, vaccine efficacy or effectiveness decreased by 10.0% (95% CI, 6.1–15.4) in people of all ages. In this meta-analysis, the studies of Ad26.COV2.S durability of protection were limited in number and sample size and hence inconsistent especially with regards to durability of protection against breakthrough infections. For example, a study by Lin and colleagues [27] found that for breakthrough infections, VE declined from 74.8% (95% CI, 72.5–76.9) at month 1 to 64.0% (95% CI, 60.3–67.4) at month 6. For hospitalization, VE remained stable, ranging from 77.7% (95% CI, 68.0–84.5) in month 1, to 81.7% (95% CI, 68.6–89.3) in month 6. A study by Rosenberg showed no time trend or waning of effectiveness for hospitalizations (RR 0.82 [95% CI, 0.63–1.07]) [9]. On the other hand, one study showed a large decline in protection against breakthrough infections for Ad26.COV2.S ranging from 64% (95% CI, 58–69) on day 14 to 36% (95% CI, 31–42) on day 172 [28]. Another showed a decline ranging from 52% (95% CI, 44–59) at day <90 from vaccination to 28% (95% CI, −8–53) [29], however, the CI was very wide due to the limited number of events. The present study confirms the durability of protection of single-dose Ad26.COV2.S against hospitalizations and severe disease.

Results from the present study are consistent with the findings from the meta-analysis for BNT162b2 and mRNA-1273 for breakthrough infections and for mRNA-1273 hospitalizations. For BNT162b2 hospitalization, the pattern of declining effectiveness for BNT162b2 from 27 February to the end of the study follow-up is consistent with the published literature. However, this study is the first to report such a large decline in effectiveness leveraging the full study period of January to September. This finding requires further investigation, especially since stratifications by age and comorbidities showed no discernible pattern to the decline in effectiveness. Moreover, the translated VE against hospitalization for BNT162b2 (January to September data) was lower than those observed in clinical trials and other real-world effectiveness studies, which may suggest a potential unobservable difference in this population. The characteristics of the populations of those vaccinated from 1 January to 27 February had substantially more comorbidities (Gagne score ≥ 6) and were older than those vaccinated from 27 February to 7 September. Furthermore, many of the early recipients of BNT162b2 were health care workers who may have been at high risk for viral exposure and transmission due to their occupation. This hypothesis, however, is not testable in insurance claims databases because occupational status is not recorded.

Four large representative studies examined the durability of effectiveness in preventing hospitalization in BNT162b2. One study showed a decline in VE from 91% (95% CI, 88–93) on day 14–120 to 77% (95% CI, 67–84) on day 121 or after [7]. A study by Tartof and colleagues showed no decline in VE against hospitalization [5]. A study from Qatar found a decline in VE against hospitalization from 96% (95% CI, 93.9–97.4) at month 1 to 55.6% (95% CI, −44.3–86.3) at month 7 or after [30]. This declining VE was based on very few events (six among cases and 11 among controls) [30] and therefore has very wide CIs. VE against hospitalization remained high at month 6 (VE 88.9% [95% CI, 52.1–97.4]). Moreover, the study by Lin and colleagues found the VE for BNT162b2 against breakthrough infections to decline from 85.5% (95% CI, 85.0–86.0) at month 1 to 67.8% (95% CI, 65.9–69.7) at month 8 [27]. The VE against hospitalization remained high and there was no discernible pattern of decline. Further population-based studies are needed to understand the drivers of this observed discrepancy in the declining VE for BNT162b2 and to evaluate the long-term durability of protection of the primary series.

Several additional limitations should be considered when interpreting our results. First, although we reduced differences between cases and controls that could affect the probability of COVID-19-related outcomes through matching, there could be remaining unmeasured effects such as occupation-related exposure. The analysis strategy maximized the number of cases by disallowing cases from serving as controls for other cases. This could have induced bias since the experience of the controls is not representative of the true experience of vaccinated individuals. However, simulations of this strategy suggest this bias is limited when event rates are low, as they were in this study. Individuals were included in this database for COVID-19-related activities; as such, the unvaccinated population was restricted to those interacting with the healthcare system for COVID-19-related concerns and thus is not representative of the general population and may have inflated VE estimates. Finally, this study relied primarily on open claims, which means other COVID-19-related medical encounters may have occurred within our population that we did not observe. However, individuals included in our study had observable vaccination records as well as healthcare utilization prior to vaccination, so observability should not be a significant limitation. Finally, this study is restricted in duration to an era before the dominance of the Omicron variant. However, the study remains relevant for understanding the natural course of VE in 2021 and will have implications for 2022 and beyond.

## 5. Conclusions

In summary, while the starting protection level provided by the primary series may differ by vaccine type and mechanism of action, this study suggested by comparing each vaccine to its own baseline protection that the three vaccines may have unique durability profiles. As the COVID-19 pandemic continues, as low to middle income countries remain largely unvaccinated and as more high-income countries implement a standard of care consisting of one or more boosters, further investigation is critical to understand the level of protection and the durability of response over longer periods, novel variants, and in response to homologous and heterologous boosting.

## Figures and Tables

**Table 1:** Demographic characteristics of vaccine recipients for Ad26.COV2.S, BNT162b2, and mRNA-1273 vaccine cohorts.

Characteristic	Ad26.COV2.S No. (%)	All BNT162b2 No. (%)	BNT162b2 No. (%) (for Primary Vaccine Regimens Using a Subset of Data Available from 27 February 2021–7 September 2021)	All mRNA-1273 No. (%)	mRNA-1273 No. (%) (for Primary Vaccine Regimens Using a Subset of Data Available from 27 February 2021–7 September 2021)
No.	1,761,498	7,903,410	6,700,042	7,352,527	6,466,763
Age, mean (SD)	50.9 (17.5)	53.1 (19.4)	50.3 (17.9)	56.6 (18.8)	55.3 (18.3)
Age group (years)					
18–24	156,961 (8.9%)	683,915 (8.7%)	651,453 (9.7%)	457,258 (6.2%)	435,240 (6.7%)
25–29	98,955 (5.6%)	451,780 (5.7%)	421,484 (6.3%)	333,920 (4.5%)	309,484 (4.8%)
30–34	111,886 (6.4%)	512,443 (6.5%)	478,469 (7.1%)	379,781 (5.2%)	351,542 (5.4%)
35–39	123,369 (7.0%)	537,280 (6.8%)	499,979 (7.5%)	407,096 (5.5%)	374,108 (5.8%)
40–44	130,393 (7.4%)	544,381 (6.9%)	504,248 (7.5%)	422,532 (5.7%)	386,331 (6.0%)
45–49	144,644 (8.2%)	573,498 (7.3%)	529,015 (7.9%)	451,982 (6.1%)	414,420 (6.4%)
50–54	181,413 (10.3%)	681,287 (8.6%)	625,690 (9.3%)	569,539 (7.7%)	526,020 (8.1%)
55–59	213,464 (12.1%)	797,898 (10.1%)	727,649 (10.9%)	699,247 (9.5%)	648,792 (10.0%)
60–64	218,591 (12.4%)	869,285 (11.0%)	787,033 (11.7%)	813,055 (11.1%)	753,929 (11.7%)
65–69	143,103 (8.1%)	662,119 (8.4%)	549,080 (8.2%)	907,660 (12.3%)	799,975 (12.4%)
70–74	104,923 (6.0%)	553,754 (7.0%)	408,479 (6.1%)	766,263 (10.4%)	642,708 (9.9%)
75–79	62,574 (3.6%)	371,754 (4.7%)	235,230 (3.5%)	485,138 (6.6%)	374,793 (5.8%)
80–84	32,566 (1.8%)	230,994 (2.9%)	127,075 (1.9%)	274,499 (3.7%)	202,833 (3.1%)
85+	38,455 (2.2%)	432,561 (5.5%)	154,822 (2.3%)	383,911 (5.2%)	246,018 (3.8%)
Sex					
Male	845,728 (48.0%)	3,286,709 (41.6%)	2,869,428 (42.8%)	3,125,010 (42.5%)	2,794,551 (43.2%)
Female	915,727 (52.0%)	4,616,322 (58.4%)	3,830,239 (57.2%)	4,227,255 (57.5%)	3,671,984 (56.8%)
Gagne combined comorbidity score					
Mean (SD)	0.41 (1.28)	0.53 (1.48)	0.41 (1.28)	0.52 (1.46)	0.48 (1.39)
Median (IQR)	0.0 (0.0)	0.0 (1.0)	0.0 (0.0)	0.0 (1.0)	0.0 (1.0)
Comorbidity score category					
<2	1,576,908 (89.5%)	6,865,542 (86.9%)	5,993,827 (89.4%)	6,375,144 (86.7%)	5,665,073 (87.6%)
2–3	126,114 (7.2%)	662,133 (8.4%)	483,613 (7.2%)	635,831 (8.6%)	533,955 (8.3%)
4–5	34,213 (1.9%)	212,538 (2.7%)	129,349 (1.9%)	199,039 (2.7%)	157,664 (2.4%)
6+	24,263 (1.4%)	163,197 (2.1%)	93,253 (1.4%)	142,513 (1.9%)	110,071 (1.7%)
Medicaid insured	154,072 (8.7%)	570,587 (7.2%)	485,282 (7.2%)	554,110 (7.5%)	475,092 (7.3%)

IQR, interquartile range; SD, standard deviation.

## Data Availability

The de-identified row-level data may be obtained through a HealthVerity data license and are not publicly available.

## Supplementary Materials

The following supporting information can be downloaded at: https://www.mdpi.com/article/10.3390/vaccines10091458/s1, Supplementary Methods; Figure S1: schematic of the study design; Figure S2: study inclusion flowchart; Figure S3: OR and 95% CI assessing durability of baseline vaccine protection against infections and hospitalizations in persons age 65 years or older; Figure S4: OR and 95% CI assessing durability of baseline vaccine protection against infections and hospitalizations in persons younger than 65 years of age; Figure S5: OR and 95% CI assessing durability of baseline vaccine protection against infections and hospitalizations in persons with comorbidity score less than 2; Figure S6: OR and 95% CI assessing durability of baseline vaccine protection against infections and hospitalizations in persons with comorbidity score greater than or equal to 2; Table S1: number of cases and matched controls by vaccine and outcome; Table S2: characteristics of cases receiving BNT162b2 in January to 27 February 2021 and 27 February 2021 to 7 September 2021; Table S3. ORs (95% CI) assessing durability of baseline vaccine protection against infections, hospitalizations, and ICU admissions separately for BNT162b2, mRNA-1273, and Ad26.COV2.S. (**a**) ORs and 95% CI for BNT162b2, mRNA-1273, and Ad26.COV2 cohorts from 27 February 2021 to 7 September 2021 requiring second dose for BNT162b2, mRNA-1273 to occur on or after 27 February 2021; (**b**) ORs and 95% CI for BNT162b2, mRNA-1273 full cohorts allowing 2nd vaccine dose to occur between 1 January 2021 and 26 February 2021 or after 26 February 2021; Table S4. Estimated VE against infections and hospitalizations by month of follow-up separately for BNT162b2, mRNA-1273, and Ad26.COV2.S vaccine cohorts from 27 February 2021 to 7 September 2021, and for BNT162b2, mRNA-1273 cohorts from 1 January 2021 to 7 September 2021 [31,32,33,34,35,36,37,38,39,40,41,42,43,44].
