# Supplementary material for: Durability of Protection Post–Primary COVID-19 Vaccination in the United States
Source: Vaccines (Basel). 2022 Sep 3;10(9):1458. doi: 10.3390/vaccines10091458 (PMC9505933; doi:10.3390/vaccines10091458)
Supplement: Supplementary file 1 [file vaccines-10-01458-s001.zip › vaccines-1794644-supplementary.pdf]

Supplementary Materials

Supplementary Methods
Figure S1. Schematic of the study design.
Figure S2. Study inclusion flowchart.
Figure S3. OR and 95% CI assessing durability of baseline vaccine protection against infections and hospitalizations in persons age 65 years or older.
Figure S4. OR and 95% CI assessing durability of baseline vaccine protection against infections and hospitalizations in persons younger than 65 years of age.
Figure S5. OR and 95% CI assessing durability of baseline vaccine protection against infections and hospitalizations in persons with comorbidity score less than 2.
Figure S6. OR and 95% CI assessing durability of baseline vaccine protection against infections and hospitalizations in persons with comorbidity score greater than or equal to 2.
Table S1. Number of cases and matched controls by vaccine and outcome.
Table S2. Characteristics of cases receiving BNT162b2 in January to 27 February 2021 and 27 February 2021 to 7 September 2021.
Table S3. ORs (95% CI) assessing durability of baseline vaccine protection against infections, hospitalizations, and ICU admissions separately for BNT162b2, mRNA-1273, and Ad26.COV2.S. (a) ORs and 95% CI for BNT162b2, mRNA-1273, and Ad26.COV2 cohorts from 27 February 2021 to 7 September 2021 requiring second dose for BNT162b2, mRNA-1273 to occur on or after 27 February 2021; (b) ORs and 95% CI for BNT162b2, mRNA-1273 full cohorts allowing 2nd vaccine dose to occur between 01 Jan 2021 and 26 Feb 2021 or after 26 Feb 2021.
Table S4. Estimated VE against infections and hospitalizations by month of follow-up separately for BNT162b2, mRNA-1273, and Ad26.COV2.S vaccine cohorts from 27 February 2021 to 7 September 2021, and for BNT162b2, mRNA-1273 cohorts from 01 January 2021 to 7 September 2021.

Supplementary Methods

Data

We analyzed de-identified person-level longitudinal data captured from medical and pharmacy claims, laboratory tests and results, and hospital chargemaster data from over 70 sources in the United States aggregated by HealthVerity. This database has been used in other COVID-19 research studies [31–34]. We drew cohorts for the current study from a broadly defined COVID-19 dataset where individuals were included based on having any documentation of COVID-19 related diagnoses, laboratory orders, procedures, treatments, or COVID-19 vaccinations. The dataset contained all major insurance types in the United States in proportions representative of the US population: Medicaid (19%), Medicare (14%), commercial (60%), and federal (5%) [21]. Race and ethnicity information was not available as they were considered personally identifiable information by HealthVerity.

For the more than 168 million individuals included in the source data, all available health data regardless of relation to COVID-19 were included. Both open (claims sent from providers to payers) and closed (adjudicated) claims were used. While open claims do not include participant enrollment (limiting certainty of observability), they capture events closer to real-time since there is no lag caused by the adjudication process. To avoid inclusion of persons whose care would not be continuously available in this database (eg, persons that do not routinely receive medical care from an included healthcare system), we required prior utilization in the two years preceding vaccination for inclusion (see Population section in main text for more details).

Exclusion criteria

We excluded individuals with 1) any positive diagnostic or antibody laboratory test or a diagnostic ICD-10 code for COVID-19 before cohort entry; 2) missing age, sex, or geographic information (three-digit zip code); 3) heterologous primary series of COVID-19 vaccines (eg, one dose of BNT162b2 followed by one dose of mRNA-1273, or vice versa); 4) more or fewer than the required number of doses (eg, a second dose of the Ad26.COV2.S vaccine); and 5) no medical claims in the 2 years before their last required vaccine dose to ensure observability of patient information.

Cases and controls

Within each of the three separate vaccine cohorts, cases were those who experienced the outcome of interest while controls were those from the same vaccine cohort who did not experience the outcome of interest. We matched each case to up to 10 controls who had entered the cohort prior to or on the calendar date of the case outcome. Cases and controls were matched on calendar time, location (three-digit zip code), sex, age (5-year category), and Gagne comorbidity score category (<2, 2–3, 4–5, 6+) using risk set sampling without replacement (Figure S1) [35,36]. The case date was defined as the date of the first occurrence of a relevant code or test result during the study period.

A COVID-19-related hospitalization was defined as an inpatient hospital encounter during which a COVID-19 ICD-10-CM code (U07.1, U07.2) or positive PCR laboratory test result was recorded. For case events, we required the initial admission date to be during our study period to further avoid hospitalizations due to infections acquired prior to full vaccination. COVID-19-related ICU admissions were defined

as an inpatient hospital encounter as described above that additionally included admission to an ICU. For both outcomes, the first of this type of event during the study period was considered the case event and the case event date was set as the hospital admission date. Controls were selected and matched as described above for breakthrough infections.

Translating ORs to VE

We estimated corresponding VE for each of the three vaccines [37–39] for COVID-19 infection and COVID-19-related hospitalization outcomes. All eligible vaccinated and unvaccinated individuals were used for these estimates. Vaccinated individuals were eligible if they met the inclusion and exclusion criteria described in the main analysis. Unvaccinated individuals were defined as those without any record of any vaccine and were excluded if they met criteria 1, 2, or 5, as described for the vaccinated cohorts in the main text. Cohort entry for unvaccinated individuals used in primary vaccine regimens using a subset of data available from 27 February 2021 to 7 September 2021, was also conducted, as that was the earliest possible cohort entry date for any vaccinated individual. For the full mRNA cohorts, cohort entry for the unvaccinated was set to 14 February 2021. For criterion 5, prior medical claims were required in the 2 years preceding cohort entry.

To estimate VE, we combined the OR for the outcomes for each of the three vaccines from the conditional logistic regression models described in the main manuscript (Table 2 in main text) with models to estimate outcome-specific baseline odds among all vaccinated individuals for each vaccine.

To estimate the baseline odds of each outcome (separately for each vaccine) we fitted a logistic regression model using all individuals vaccinated with the vaccine of interest who also met all other inclusion and exclusion criteria. We used this fitted logistic regression equation to find the odds of infection among all vaccinated individuals in the first month of follow-up (baseline). To harmonize baseline odds to the unvaccinated population, we adjusted the model for age, sex, comorbidity score, and geographic location (state) according to the unvaccinated population demographics. Specifically, we categorized age (to 18–28, …, 89–98 categories) and comorbidity score (to –2–0, 0–2, 2–5, 5–25 categories) and calculated the proportion of unvaccinated individuals in each age, comorbidity score, sex, and state category. We used these categorical distributions to marginalize the estimated conditional probability from the fitted logistic regression equation to calculate the weighted probability of getting an outcome of interest in the first month, where the weighting was according to the unvaccinated population demographics. Under assumptions of independence the weighted probability of getting an outcome:

$$P(Y=1) = \sum_{i,j,l,k} P\left(Y=1 \middle| age_i, sex_j, c\_score_l, state_k\right) P(age_i) P\left(sex_j\right) P(c\_score_l) P(state_k),$$

where $Y = 1$ indicates that the outcome is observed.

Finally, we calculated the baseline odds as the weighted probability of having the outcome of interest in the first month over one minus that probability:

$$odds_{baseline} = \frac{P(Y=1)}{1 - P(Y=1)}.$$

Next, we multiplied the baseline odds of outcome (among all vaccinated with the vaccine of interest) by the OR for the second, third, fourth, fifth, and sixth months of follow-up from the conditional logistic

regression models used in the main analysis to approximate the odds of a positive test in the second, third, fourth, fifth, and sixth months respectively:

$$odds_{month_k} = odds_{baseline} OR_{month_k}.$$

We used the odds of outcome in each month to calculate the probability of that outcome in the one-month period to approximate the incidence proportion for each month for the outcome of interest among those who had received the vaccine of interest:

$$IP_{vaccinated_{month_k}} = \frac{odds_{month_k}}{1+odds_{month_k}}.$$

We next estimated the incidence proportion among the unvaccinated. First, we calculated IP for each calendar month in follow-up (February–August). Then, we calculated the proportion of cases in each month of follow-up by the calendar month in the vaccinated cohort (separately for each vaccine) and used these proportions as weights to calculate IP in the unvaccinated for each month of follow-up separately:

$$IP_{unvaccinated_{month_k}} = w_{Feb}^{month_k} IP_{unvaccinated_{Feb}} + \cdots + w_{Aug}^{month_k} \ IP_{unvaccinated_{Aug}},$$

where $w_J^{month_k}$ – is the proportion of cases in month k of follow-up that happened in calendar month J. For example, consider Ad26.COV2.S vaccinated cohort infections outcome. There were 15% of cases in July and 85% in August in the month 5 since follow-up. Then, we calculate the IP for unvaccinated for month 5 of follow-up as

$$IP_{unvaccinated_{month_5}} = 0.15 \ IP_{unvaccinated_{July}} + 0.85 \ IP_{unvaccinated_{Aug}}.$$

We calculated the VE for each month as one minus the ratio of incidence proportions for the vaccinated as compared to the unvaccinated:

$$VE_{month_k} = 1 - \frac{IP_{vaccinated_{month_k}}}{IP_{unvaccinated_{month_k}}}.$$

Confidence intervals (95%) for VE estimates were obtained by bootstrapping resampling over 1000 replicates [40].

Sensitivity analyses

To assess durability of protection in groups at relatively higher or lower risk, we stratified cohorts by age and comorbidity score. Due to the low numbers of ICU admission cases, we restricted all sensitivity analyses to infections and hospitalizations. For age-stratified analyses, we repeated the original analysis using the same procedures and methods and built models separately for study participants 65 years and older and younger than 65 years of age. Similarly, for comorbidity-stratified analyses, we repeated our analyses for study participants with a comorbidity score of less than two and a comorbidity score of two or greater.

Vaccine effectiveness (VE)

VE for infection and hospitalizations remained strong for all three vaccines throughout the follow-up period (Table 3). However, these results differed for BNT162b2, and mRNA-1273 when using the full available data (1 January 2021 to 7 September 2021) or when aligning the cohort entry time to match the entry time of Ad26.COV2.S (27 February 2021) especially for hospitalization. Leveraging the full available data, for infections, VE in the first month of follow-up was 0.74, 0.88, and 0.92 for Ad26.COV2.S, BNT162b2, and mRNA-1273 vaccines, respectively. Ad26.COV2.S showed stable VE over time. The mRNA vaccines had decreased VE by the final month of follow-up with BNT162b2 decreasing to 0.71 and mRNA-1273 decreasing to 0.82 in month 6+. Similar trends were observed when aligning the cohort entry date to 27 February 2021.

For COVID-19-related hospitalizations, using all available data, VE in the first month of follow-up was 0.81, 0.89, and 0.94 for Ad26.COV2.S, BNT162b2, and mRNA-1273 vaccines, respectively. In month 5+, Ad26.COV2.S VE against hospitalization was 0.76, and for BNT162b2, and mRNA-1273 in the 6+ month of follow-up VE against hospitalization was 0.52 and 0.90 respectively. When aligning the cohort entry date to 27 February 2021, for BNT162b2, and mRNA-1273, the waning in VE for BNT162b2 became less pronounced. Trends for mRNA-1273 VE remained similar to those previously observed.

These results confirm and extend estimations of peak VE (ie, in the first month after full vaccination) for all three vaccines separately against infections and severe disease although the calculated VE against hospitalization for BNT162b2 was lower than the VE reported in clinical trials and other real-world effectiveness studies. The observed waning in VE against hospitalization for BNT162b2 requires further discussion. The apparent clear decline in VE over time is observed when the study cohort includes subjects vaccinated in January and February 2021. This may be driven, at least in part, by waning immune responses in older and more comorbid patients (Table 1; Table S2). Moreover, subgroups may experience differential waning. For example, vaccine administration was initially prioritized for health care workers and residents of long-term care facilities and these individuals might account for the differences in durability estimates observed when those vaccinated in January and February 2021 were included in the BNT162b2 cohort. These individuals may have had great SARS-CoV-2 exposure, more frequent testing, or different immune responses (in the case of long-term care residents) as compared to other individuals. Two distinct factors may contribute to the durability of protection or declining effectiveness of a given vaccine. One is the waning immunity against a stable pathogen, due to declines in antibody levels, B-cell memory, and/or T-cell populations. Second is the emergence of novel variants of the pathogen. Without access to pathogen strain data and repeated measures of immune responses, large population-based observational studies may not be able to directly disentangle the effects of these two factors. Further investigation is needed to fully understand the complete durability profile of each of the vaccines.

Our study population is consistent with previously published trends in one of the largest samples to date, including approximately 10% of the fully vaccinated individuals in the United States during our study period [20]. This is of particular importance for the evaluation of the Ad26.COV2.S vaccine, which has generally been assessed with much smaller samples relative to the mRNA vaccines or not at all. Our methods for translating OR to VE come with several limitations. First, we estimated incidence proportion from large cohorts of vaccinated and unvaccinated individuals where absolute case numbers likely represent underestimates, in part due to the potential for asymptomatic and unconfirmed infections [41–43]. While this is not an issue if this is equally true for both vaccinated and unvaccinated groups, if providers

tested or hospitalized individuals differently based on vaccine status, this may result in bias. Second, many COVID-19-related encounters in the United States, including vaccination and tests, occurred in settings where they were not billed to any payer (eg, mass vaccination clinics) and thus cannot be observed in claims. This can affect the results primarily via misclassification of unvaccinated individuals. This issue would be expected to result in an underestimate of VE [4,44]. Finally, the cohorts differ by the timing of events, and relatedly, their demographic makeup. As described above, we attempted to harmonize the baseline odds according to the demographic composition of the unvaccinated cohort. However, demographic differences persist and may limit the comparability of VE across vaccines.

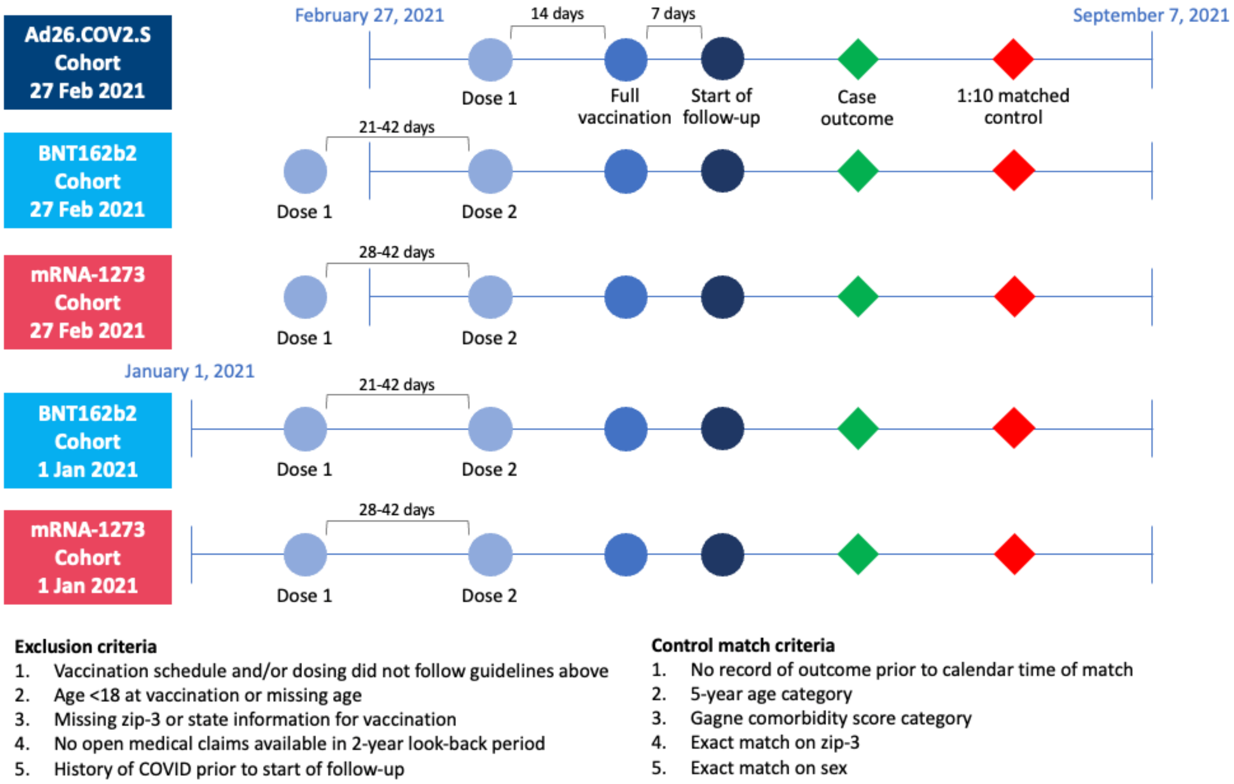

Figure S1. Schematic of the study design.

Note: Cases and controls were matched on outcome calendar time. Follow-up time was determined by looking back to the start of follow-up, which varied for cases and matched controls. Cohorts for Ad26.COV2.S required first (and only) dose and for mRNA-1273 and BNT162b2 required the second dose to be on Feb. 27 or after. Additional cohorts for BNT162b2 and mRNA-1273 allowed the second dose to be administered before Feb. 27.

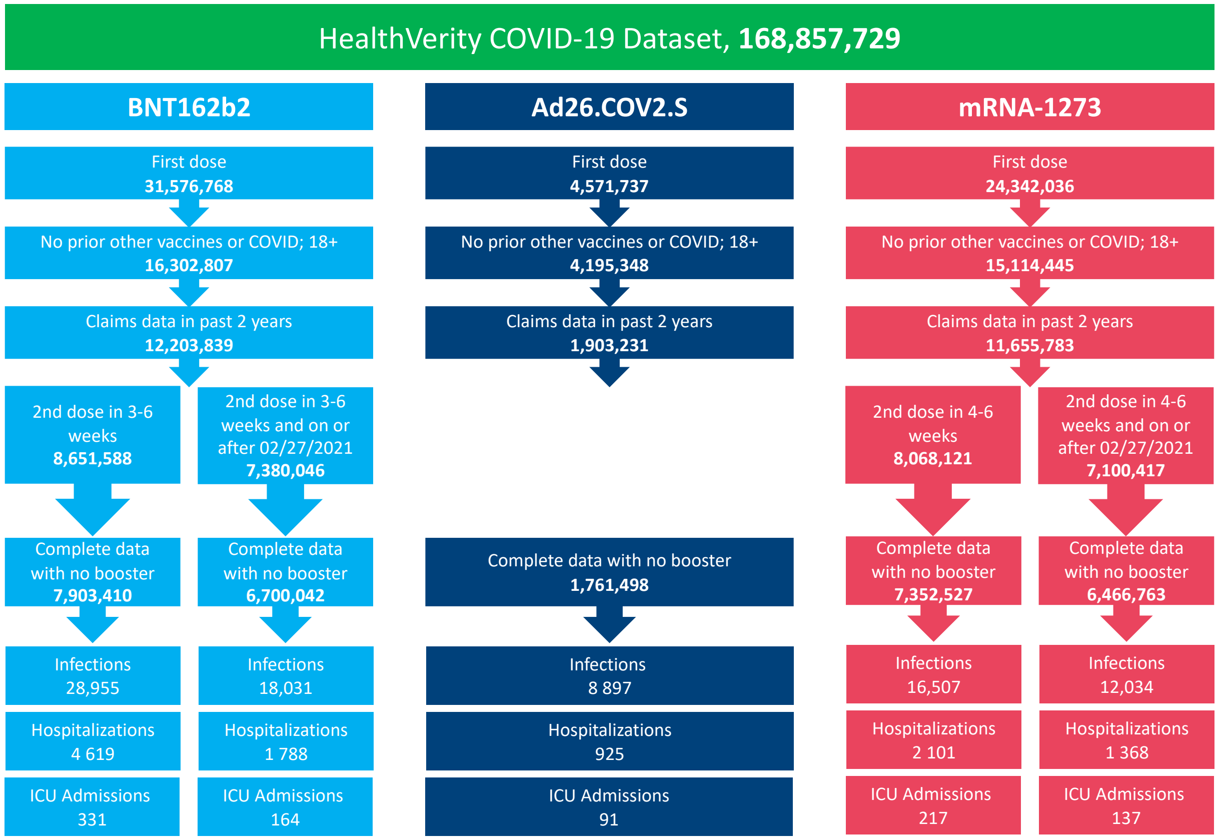

Figure S2. Study inclusion flowchart.

ICU, intensive care unit.

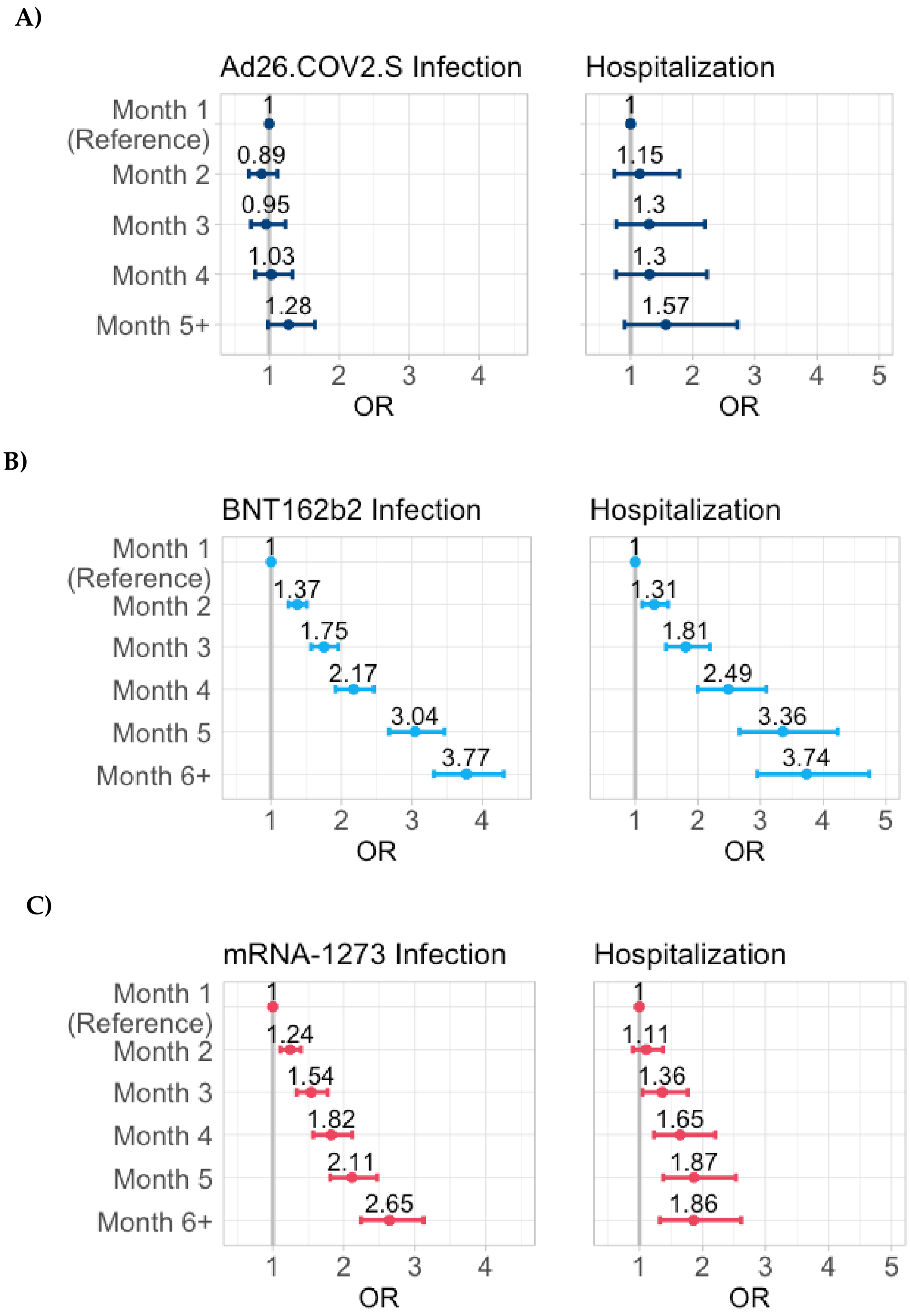

Figure S3. OR and 95% CI assessing durability of baseline vaccine protection against infections and hospitalizations in persons age 65 years or older.

(**A**) Ad26.COV2.S, data available 27 February–7 September 2021, (**B**) BNT162b2 data available 1 January–7 September 2021, (**C**) mRNA-1273 data available 1 January–7 September 2021. CI, confidence interval; OR, odds ratio.

A)

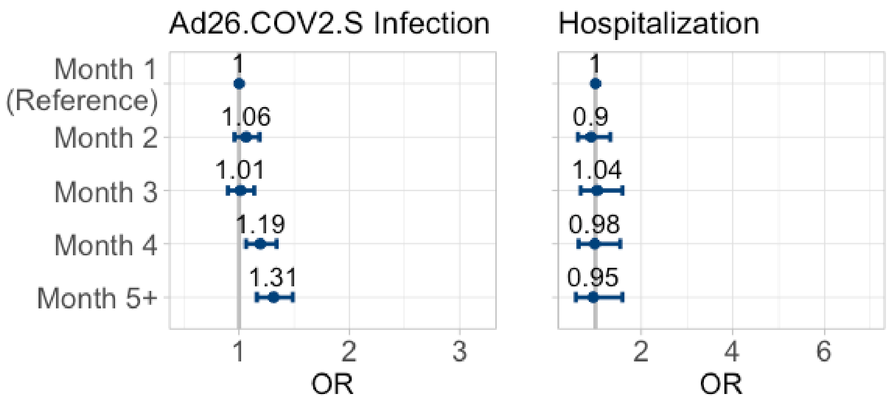

B)

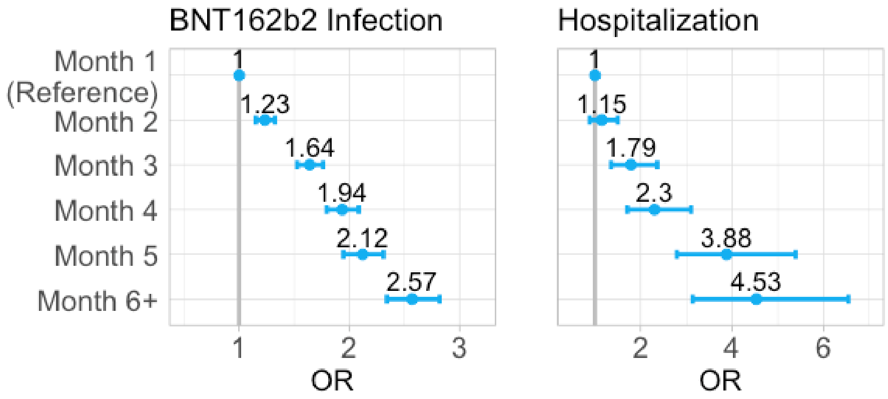

C)

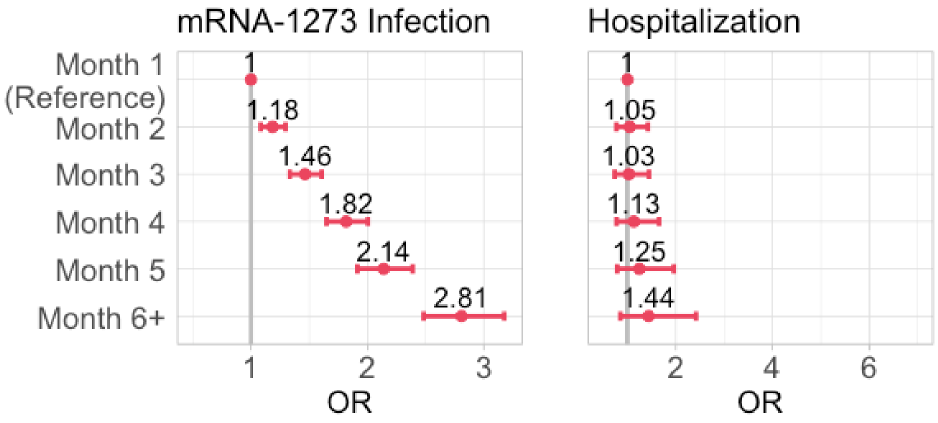

Figure S4. OR and 95% CI assessing durability of baseline vaccine protection against infections and hospitalizations in persons younger than 65 years of age.

A) Ad26.COV2.S data available from 27 February–7 September 2021, B) BNT162b2 data available from 1 January–7 September 2021, C) mRNA-1273 data available from 1 January–7 September 2021. CI, confidence interval; OR, odds ratio.

A)

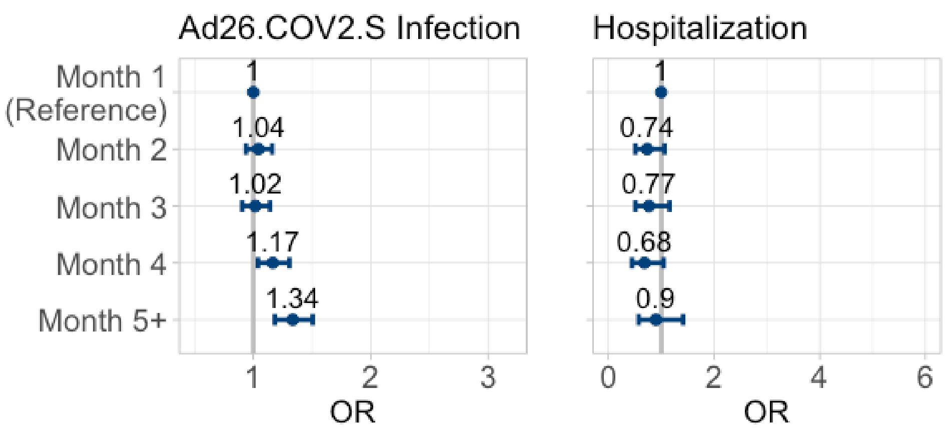

B)

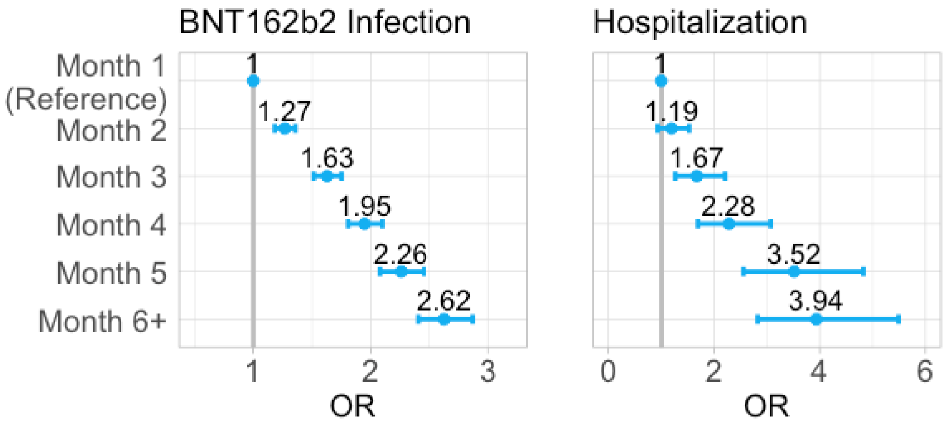

C)

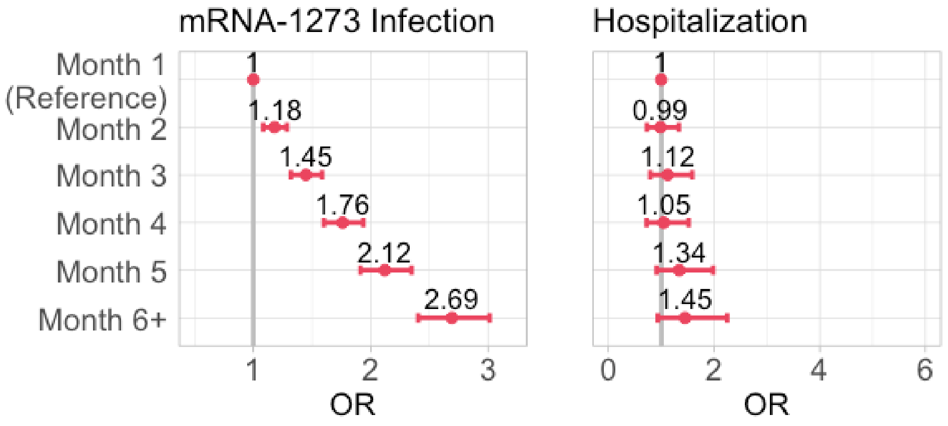

Figure S5. OR and 95% CI assessing durability of baseline vaccine protection against infections and hospitalizations in persons with comorbidity score less than 2.

A) Ad26.COV2.S data available from 27 February–7 September 2021, B) BNT162b2 data available from 1 January–7 September 2021, C) mRNA-1273 data available from 1 January–7 September 2021. CI, confidence interval; OR, odds ratio.

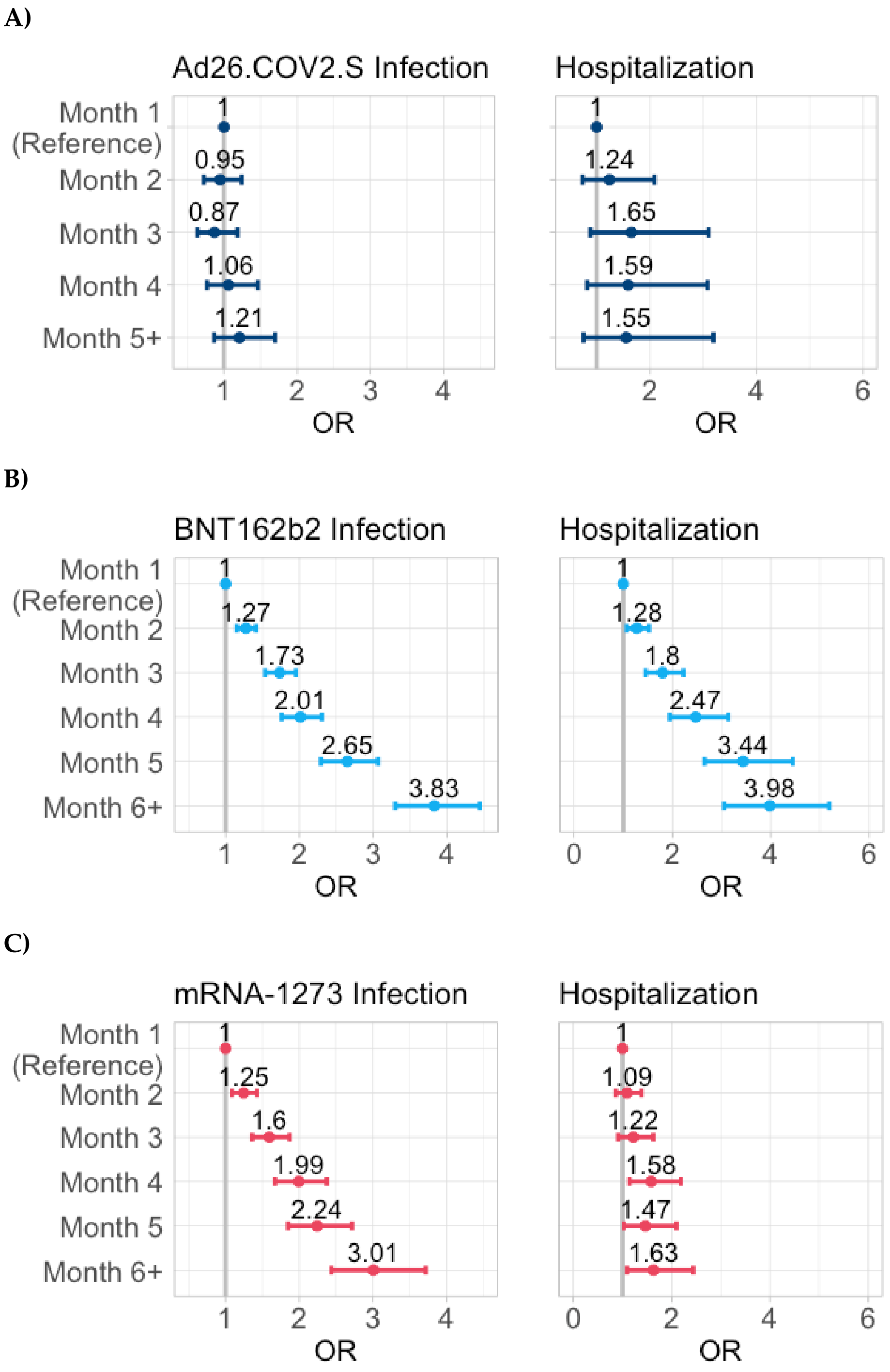

Figure S6. OR and 95% CI assessing durability of baseline vaccine protection against infections and hospitalizations in persons with comorbidity score greater than or equal to 2.

A) Ad26.COV2.S (Data available from 27 February–7 September 2021) B) BNT162b2 (Data available from 1 January–7 September 2021) C) mRNA-1273 (Data available from 1 January–7 September 2021). CI, confidence interval; OR, odds ratio.

Table S1. Number of cases and matched controls by vaccine and outcome.

Vaccine	Outcome	Cases (N)	Matched controls (N)
Ad26.COV2.S	Infection	7945	67,771
	Hospitalization	823	5991
	ICU admission	82	595
All BNT162b2	Infection	27,797	261,200
	Hospitalization	4525	42,233
	ICU admission	320	2989
BNT162b2 (for primary vaccine regimens using a subset of data available from 27 February 2021–7 September 2021)	Infection	17,774	167,218
	Hospitalization	1736	15,223
	ICU admission	160	1410
All mRNA-1273	Infection	15,694	148,709
	Hospitalization	2070	19,176
	ICU admission	212	2057
mRNA-1273 (for primary vaccine regimens using a subset of data available from 27 February 2021–7 September 2021)	Infection	11,883	112,583
	Hospitalization	1336	12,112
	ICU admission	134	1306

ICU, intensive care unit.

Table S2. Characteristics of cases receiving BNT162b2 from 1 January to 27 February 2021 and 27 February 2021 to 7 September 2021.

	Infections		Hospitalizations	
	**Cohort vaccinated between 1 Jan 2021 and 26 Feb 2021 (N=10,051)**	**Cohort vaccinated on or after 27 Feb 2021 (N=17,774)**	**Cohort vaccinated between 1 Jan 2021 and 26 Feb 2021 (N=2791)**	**Cohort vaccinated on or after 27 Feb 2021 (N=1736)**
**Age**				
Mean (SD)	72.1 (19.0)	51.3 (18.2)	80.3 (13.8)	67.7 (17.1)
Median [Min, Max]	75.0 [18.0, 94.0]	52.0 [18.0, 94.0]	81.0 [19.0, 94.0]	69.0 [18.0, 94.0]
Missing	0 (0%)	1 (0.0%)	0 (0%)	0 (0%)
**Age group**				
18–24	198 (2.0%)	1517 (8.5%)	3 (0.1%)	31 (1.8%)
25–29	156 (1.6%)	1045 (5.9%)	8 (0.3%)	20 (1.2%)
30–34	210 (2.1%)	1275 (7.2%)	13 (0.5%)	39 (2.2%)
35–39	243 (2.4%)	1361 (7.7%)	16 (0.6%)	38 (2.2%)
40–44	271 (2.7%)	1373 (7.7%)	17 (0.6%)	37 (2.1%)
45–49	283 (2.8%)	1440 (8.1%)	30 (1.1%)	63 (3.6%)
50–54	394 (3.9%)	1625 (9.1%)	44 (1.6%)	114 (6.6%)
55–59	495 (4.9%)	1877 (10.6%)	82 (2.9%)	143 (8.2%)
60–64	639 (6.4%)	1965 (11.1%)	130 (4.7%)	194 (11.2%)
65–69	872 (8.7%)	1462 (8.2%)	199 (7.1%)	221 (12.7%)
70–74	1218 (12.1%)	1131 (6.4%)	308 (11.0%)	227 (13.1%)
75–79	1205 (12.0%)	715 (4.0%)	395 (14.2%)	188 (10.8%)
80–84	984 (9.8%)	423 (2.4%)	349 (12.5%)	157 (9.0%)
85+	2883 (28.7%)	565 (3.2%)	1197 (42.9%)	264 (15.2%)
**Sex**				
Female	6268 (62.4%)	10,380 (58.4%)	1637 (58.7%)	882 (50.8%)
Male	3783 (37.6%)	7394 (41.6%)	1154 (41.3%)	854 (49.2%)
**Gagne**				
Mean (SD)	2.21 (2.82)	0.771 (1.82)	3.04 (2.96)	2.44 (3.08)
Median [Min, Max]	1.00 [−1.00, 18.0]	0 [−2.00, 18.0]	2.00 [−1.00, 17.0]	1.00 [−1.00, 18.0]
**Gagne group**				
<2	5304 (52.8%)	14,528 (81.7%)	1050 (37.6%)	875 (50.4%)
2–3	2124 (21.1%)	1961 (11.0%)	711 (25.5%)	375 (21.6%)
4–5	1240 (12.3%)	671 (3.8%)	475 (17.0%)	206 (11.9%)
6+	1383 (13.8%)	614 (3.5%)	555 (19.9%)	280 (16.1%)
**Medicaid**				
Yes	2267 (22.6%)	2502 (14.1%)	793 (28.4%)	336 (19.4%)
No	7784 (77.4%)	15,272 (85.9%)	1998 (71.6%)	1400 (80.6%)

SD, standard deviation.

Table S3. ORs (95% CI) assessing durability of baseline vaccine protection against infections, hospitalizations, and ICU admissions separately for BNT162b2, mRNA-1273, and Ad26.COV2.S. (**a**) ORs and 95% CI for BNT162b2, mRNA-1273, and Ad26.COV2 cohorts from 27 February 2021 to 7 September 2021 requiring second dose for BNT162b2, mRNA-1273 to occur on or after 27 February 2021; (**b**) ORs and 95% CI for BNT162b2, mRNA-1273 full cohorts allowing 2nd vaccine dose to occur between 01 Jan 2021 and 26 Feb 2021 or after 26 Feb 2021.

**Vaccine** (time frame)	Breakthrough Infection OR * (95% CI)	Hospitalization OR * (95% CI)	ICU Admissions OR *,† (95% CI)
**Ad26.COV2.S** (Data available from 27 February 2021–7 September 2021)			
Month 1	1 (Reference)	1 (Reference)	1 (Reference)
Month 2	1.03 (0.94–1.14)	1.01 (0.76–1.34)	0.96 (0.39–2.41)
Month 3	0.99 (0.89–1.11)	1.15 (0.83–1.60)	1.89 (0.65–5.51)
Month 4	1.16 (1.04–1.29)	1.11 (0.79–1.56)	1.40 (0.43–4.55)
Month 5 or after	1.31 (1.18–1.47)	1.25 (0.86–1.80)	..
**BNT162b2** (Data available from 27 February 2021–7 September 2021)			
Month 1	1 (Reference)	1 (Reference)	1 (Reference)
Month 2	1.21 (1.13–1.31)	1.10 (0.88–1.37)	0.89 (0.52–1.51)
Month 3	1.57 (1.46–1.70)	1.45 (1.13–1.86)	0.63 (0.31–1.27)
Month 4	1.88 (1.74–2.04)	1.86 (1.43–2.43)	1.62 (0.76–3.45)
Month 5 or after	2.20 (2.01–2.40)	2.38 (1.79–3.17)	..
**mRNA-1273** (Data available from 27 February 2021–7 September 2021)			
Month 1	1 (Reference)	1 (Reference)	1 (Reference)
Month 2	1.13 (1.04–1.23)	1.03 (0.82–1.28)	0.67 (0.39–1.16)
Month 3	1.38 (1.26–1.51)	1.04 (0.80–1.34)	0.54 (0.26–1.12)
Month 4	1.77 (1.61–1.94)	1.17 (0.89–1.54)	1.03 (0.44–2.41)
Month 5 or after	2.07 (1.87–2.29)	1.32 (0.98–1.79)	..
**BNT162b2** (Data available from 1 January 2021–7 September 2021)			
Month 1	1 (Reference)	1 (Reference)	1 (Reference)
Month 2	1.28 (1.21–1.36)	1.26 (1.10–1.44)	1.20 (0.80–1.80)
Month 3	1.68 (1.58–1.78)	1.81 (1.55–2.12)	0.73 (0.44–1.21)
Month 4	1.99 (1.87–2.12)	2.45 (2.05–2.92)	1.36 (0.80–2.30) †
Month 5	2.37 (2.21–2.54)	3.54 (2.93–4.27)	..
Month 6 or after	2.93 (2.72–3.15)	3.97 (3.26–4.83)	..
**mRNA-1273** (Data available from 1 January 2021–7 September 2021)			
Month 1	1 (Reference)	1 (Reference)	1 (Reference)
Month 2	1.21 (1.13–1.30)	1.08 (0.91–1.29)	0.62 (0.40–0.97)
Month 3	1.49 (1.38–1.61)	1.23 (1.01–1.51)	0.73 (0.43–1.23)
Month 4	1.83 (1.68–1.98)	1.41 (1.12–1.77)	1.17 (0.64–2.13) †
Month 5	2.15 (1.97–2.35)	1.62 (1.27–2.07)	..
Month 6 or after	2.76 (2.51–3.04)	1.66 (1.26–2.19)	..

CI, confidence interval; ICU, intensive care unit; OR, odds ratio. * ORs are matched and conditioned on age group, sex, Gagne comorbidity index score category, three-digit zip, and calendar date. † ICU outcome final time category is month 4 or after.

Table S4. Estimated VE against infections and hospitalizations by month of follow-up separately for BNT162b2, mRNA-1273, and Ad26.COV2.S vaccine cohorts from 27 February 2021 to 7 September 2021, and for BNT162b2, mRNA-1273 cohorts from 01 January 2021 to 7 September 2021.

**Vaccine** (time frame)	**Breakthrough Infection VE (95% CI)**	**Hospitalization VE (95% CI)**
**Ad26.COV2.S** (Data available from 27 February 2021–7 September 2021)		
Month 1	0.74 (0.72–0.75)	0.81 (0.76–0.82)
Month 2	0.68 (0.63–0.70)	0.75 (0.64–0.81)
Month 3	0.75 (0.70–0.76)	0.73 (0.61–0.80)
Month 4	0.74 (0.70–0.75)	0.77 (0.66–0.83)
Month 5 or after	0.74 (0.70–0.76)	0.77 (0.64–0.83)
**BNT162b2** (Data available from 27 February 2021–7 September 2021)		
Month 1	0.89 (0.88–0.89)	0.92 (0.91–0.93)
Month 2	0.86 (0.84–0.87)	0.90 (0.87–0.92)
Month 3	0.86 (0.84–0.87)	0.87 (0.84–0.90)
Month 4	0.86 (0.84–0.86)	0.86 (0.82–0.89)
Month 5 or after	0.84 (0.82–0.85)	0.84 (0.79–0.88)
**mRNA-1273** (Data available from 27 February 2021–7 September 2021)		
Month 1	0.92 (0.91–0.92)	0.94 (0.93–0.95)
Month 2	0.90 (0.89–0.90)	0.93 (0.91–0.94)
Month 3	0.90 (0.89–0.91)	0.93 (0.90–0.94)
Month 4	0.89 (0.88–0.90)	0.93 (0.91–0.95)
Month 5 or after	0.88 (0.87–0.89)	0.93 (0.91–0.95)
**BNT162b2** (Data available from 1 January 2021–7 September 2021)		
Month 1	0.88 (0.87–0.88)	0.89 (0.88–0.90)
Month 2	0.84 (0.83–0.84)	0.86 (0.84–0.88)
Month 3	0.79 (0.78–0.80)	0.75 (0.71–0.79)
Month 4	0.78 (0.77–0.80)	0.64 (0.58–0.71)
Month 5	0.75 (0.73–0.76)	0.52 (0.44–0.61)
Month 6 or after	0.71 (0.69–0.73)	0.52 (0.43–0.61)
**mRNA-1273** (Data available from 1 January 2021–7 September 2021)		
Month 1	0.92 (0.91–0.92)	0.94 (0.93–0.95)
Month 2	0.89 (0.88–0.90)	0.93 (0.91–0.94)
Month 3	0.87 (0.86–0.88)	0.90 (0.88–0.92)
Month 4	0.86 (0.85–0.87)	0.90 (0.87–0.92)

Month 5	0.85 (0.83–0.86)	0.89 (0.86–0.92)
Month 6 or after	0.82 (0.80–0.83)	0.90 (0.87–0.92)

VE, vaccine effectiveness.
